# A Novel Glucose Metabolism-Related Gene Signature for Overall Survival Prediction in Patients with Glioblastoma

**DOI:** 10.1155/2021/8872977

**Published:** 2021-01-22

**Authors:** Chaocai Zhang, Minjie Wang, Fenghu Ji, Yizhong Peng, Bo Wang, Jiannong Zhao, Jiandong Wu, Hongyang Zhao

**Affiliations:** ^1^Department of Neurosurgery, Hainan General Hospital/Hainan Affiliated Hospital of Hainan Medical University, Haikou 570311, China; ^2^Department of Neurosurgery, Union Hospital, Tongji Medical College, Huazhong University of Science and Technology, Wuhan 430022, China; ^3^School of Electronic Information and Communications, Huazhong University of Science and Technology, Wuhan 430074, China; ^4^Department of Orthopaedics, Union Hospital, Tongji Medical College, Huazhong University of Science and Technology, Wuhan 430022, China; ^5^Department of Central Laboratory, Hainan General Hospital/Hainan Affiliated Hospital of Hainan Medical University, Haikou 570311, China; ^6^Department of Neurosurgery, Suzhou Municipal Hospital, Nanjing Medical College, Jiangsu 215000, China

## Abstract

**Introduction:**

Glioblastoma (GBM) is one of the most frequent primary intracranial malignancies, with limited treatment options and poor overall survival rates. Alternated glucose metabolism is a key metabolic feature of tumour cells, including GBM cells. However, due to high cellular heterogeneity, accurately predicting the prognosis of GBM patients using a single biomarker is difficult. Therefore, identifying a novel glucose metabolism-related biomarker signature is important and may contribute to accurate prognosis prediction for GBM patients.

**Methods:**

In this research, we performed gene set enrichment analysis and profiled four glucose metabolism-related gene sets containing 327 genes related to biological processes. Univariate and multivariate Cox regression analyses were specifically completed to identify genes to build a specific risk signature, and we identified ten mRNAs (B4GALT7, CHST12, G6PC2, GALE, IL13RA1, LDHB, SPAG4, STC1, TGFBI, and TPBG) within the Cox proportional hazards regression model for GBM.

**Results:**

Depending on this glucose metabolism-related gene signature, we divided patients into high-risk (with poor outcomes) and low-risk (with satisfactory outcomes) subgroups. The results of the multivariate Cox regression analysis demonstrated that the prognostic potential of this ten-gene signature is independent of clinical variables. Furthermore, we used two other GBM databases (Chinese Glioma Genome Atlas (CGGA) and REMBRANDT) to validate this model. In the functional analysis results, the risk signature was associated with almost every step of cancer progression, such as adhesion, proliferation, angiogenesis, drug resistance, and even an immune-suppressed microenvironment. Moreover, we found that IL31RA expression was significantly different between the high-risk and low-risk subgroups.

**Conclusion:**

The 10 glucose metabolism-related gene risk signatures could serve as an independent prognostic factor for GBM patients and might be valuable for the clinical management of GBM patients. The differential gene IL31RA may be a potential treatment target in GBM.

## 1. Introduction

Glioblastoma (GBM), which has an annual incidence of 3.22 per 100,000, remains the most common and aggressive primary adult brain tumour [1, 2]. Standard therapeutic regimens include maximal safety surgical resection, followed by radiotherapy and chemotherapy. Moreover, targeted therapy, immunotherapy, and tumour treating fields (TTFs) are also widely used in the treatment of GBM [3, 4]. Despite applying the best standard of care, patients diagnosed with GBM usually face a dismal prognosis, with a survival time of fewer than two years for most patients [5]. More recently, many types of research have shown that altered metabolism in cancer cells is crucial for cancer growth and progression [6–8]. Among them, studies on glucose metabolism have attracted most attention and mainly involve four aspects: the tricarboxylic acid cycle (TCA cycle), glycolysis, gluconeogenesis, and glycogen synthesis [9–11]. The TCA cycle plays a central metabolic role in ATP production and is frequently dysregulated in cancer [12]. Aerobic glycolysis (Warburg's effect), one of the hallmarks of cancer, indicates that cancer cells produce lactate after absorbing glucose as a substrate for oxidative phosphorylation, even under normoxic conditions [13]. Gluconeogenesis can antagonize aerobic glycolysis in cancer via several enzymes, which also play a role in signalling transduction, cell proliferation, and the stemness of cancer cells [10]. Glycogen maintains glucose homeostasis and contributes to key functions related to aggressiveness and survival of cancer cells [11]. In GBM, the number of genes associated with glycolysis has been suggested to correlate with tumour proliferation, invasion, angiogenesis, and chemotherapy/radiotherapy resistance [14–17]. In addition, previous research has revealed a correlation between GBM glycolysis and clinical outcomes [18]. However, glucose metabolism-related gene signatures, which may more effectively predict patient prognosis, are still lacking in GBM.

In this study, we first identified all glucose metabolism-related gene sets. Then, gene set enrichment analysis (GSEA) was performed to screen out the hallmark gene sets in 167 GBM patients with entire mRNA expression data from The Cancer Genome Atlas (TCGA) database. We described 327 mRNAs significantly related to glucose metabolism and established a ten-gene risk signature that can forcefully predict patient outcomes. Notably, the glucose metabolism-related risk signature could independently determine patients in the high-risk group with poor prognosis. Finally, we further explored the underlying mechanisms and the differentially expressed genes between two groups mentioned above, and the results demonstrated that the risk signature was related to almost every step of cancer progression, such as adhesion, proliferation, angiogenesis, drug resistance, and even an immune-suppressed microenvironment. Moreover, we found that IL31RA expression was significantly different between the two groups.

## 2. Materials and Methods

### 2.1. Clinical Information and Genome Expression Data of the Samples

The transcriptional profile and clinical data of patients with low-grade glioma (LGG) and GBM were downloaded from the TCGA database online (https://cancergenome.nih.gov/). Clinical data, which contains the total number of patients (*n* = 696, including 529 LGG patients and 167 GBM patients), gender, age, Karnofsky's Performance Status (KPS) score, radiotherapy, chemotherapy, IDH status, and MGMT promoter methylation status, was collected for the study. Validation reports from the Repository for Molecular Brain Neoplasia Data (REMBRANDT, microarray) and Chinese Glioma Genome Atlas (CGGA, microarray) datasets were downloaded from GlioVis (http://gliovis.bioinfo.cnio.es/).

### 2.2. GSEA

Gene set enrichment analysis (GSEA) was used to examine whether the identified sets of genes demonstrated significant differences between the two groups [19]. The expression levels of all mRNAs in LGG and GBM were analysed using GSEA 4.0.3. Normalized *p* values (*p* < 0.05) and normalized enrichment scores (NESs) were referenced to select functions to investigate in further analysis. Gene set variation analysis (GSVA) was performed to study biological processes and Kyoto Encyclopedia of Genes and Genomes (KEGG) pathways associated with the glucose metabolism-related risk signature [20]. We used the R package “limma” to select ten associated gene sets with differences between the high-risk group and the low-risk group in the TCGA dataset (GBM HGU133A), and an adjusted *p* value < 0.05 was considered statistically significant.

### 2.3. Prognostic Potential Analysis

The associations between the mRNA expression level and patient overall survival (OS) were calculated using a univariate Cox model. The mRNAs with *p* values less than 0.05 were considered statistically significant using univariate Cox analysis. Afterward, a multivariable Cox analysis was used to evaluate the weight of mRNAs as independent predictors of survival. To reduce the test error, significant factors in the univariate analysis were selected for the multivariate analysis. These analyses were completed through the R package “survival.”

### 2.4. Statistical Analysis

The candidate genes were classified into risk (hazard ratio (HR) > 1) and protective (0 < HR < 1) types. Based on the multivariate Cox regression analysis results, a prognostic risk score formula was established using a linear combination of the expression levels multiplied by the regression coefficients. The risk score formula is indicated as follows: risk score = the expression of gene 1 × *β*1 + the expression of gene 2 × *β*2 + ⋯+the expression of gene *n* × *βn*. We divided 167 patients with GBM into high-risk and low-risk subgroups using the median risk score as the cutoff value. Kaplan-Meier (KM) curves and the log-rank test were used to validate the prognostic potential and significance of the risk score. We used Student's *t*-test to compare the differential expression of the optimal genes between LGG and GBM tissues. All of the statistical analyses were completed using SPSS 19.0 and R 3.6.3 software. The chi-square test was performed to assess the relationships between the risk score and clinical variables, and a Bonferroni correction was used to adjust the threshold of the significance of *p* values within multigroup comparisons [21].

### 2.5. Exploration of the Differentially Expressed Genes of the Signature

Median risk scores were used to divide patients into a high-risk group and a low-risk group. Differential gene expression analysis of the two groups was carried out using the EdgeR method, and a volcano map was drawn. We downloaded the transcriptome data and clinical data of glioma patients from the TCGA database (https://cancergenome.nih.gov) and CGGA database (http://www.cgga.org.cn/). Survival times and survival statuses were extracted. A KM curve was used to analyse survival differences between patients with high and low expression levels of differentially expressed genes. We also detected the immunohistochemistry of the differentially expressed genes in the tumour tissues and normal brain tissues of 20 glioma patients.

### 2.6. Immunohistochemistry

The slices (paraffin sections) of normal brain tissues and tumour tissues were used for immunohistochemistry analysis. Briefly, the slices were dewaxed, gradient dehydrated with alcohol, washed with standard method, repaired with water bath in antigen repairing solution and cooled with tap water. Then, the slices were blocked by normal goat serum solution (SAP-9100, ZSGB-BIO, Beijing, China). Then, the slices were dealt with the anti-IL31RA (K008474P, Solarbio, Beijing, China) and goat anti-rabbit IgG, and the slices were washed with standard methods, respectively. After that, the slices were dripped with Streptomyces ovalbumin protein labelled with horseradish peroxidase, and the slices were color developed with DAB (ZLI-9017, ZSGB-BIO, Beijing, China). Hematoxylin (ZLI-9610, ZSGB-BIO, Beijing, China) was used for counterstaining. Finally, the pathologist observed and interpreted the stained tissues under a light microscope.

## 3. Results

### 3.1. Glycolysis and Gluconeogenesis-Related Gene Set Differences Significant between LGG and GBM Samples

**Figure 1:**
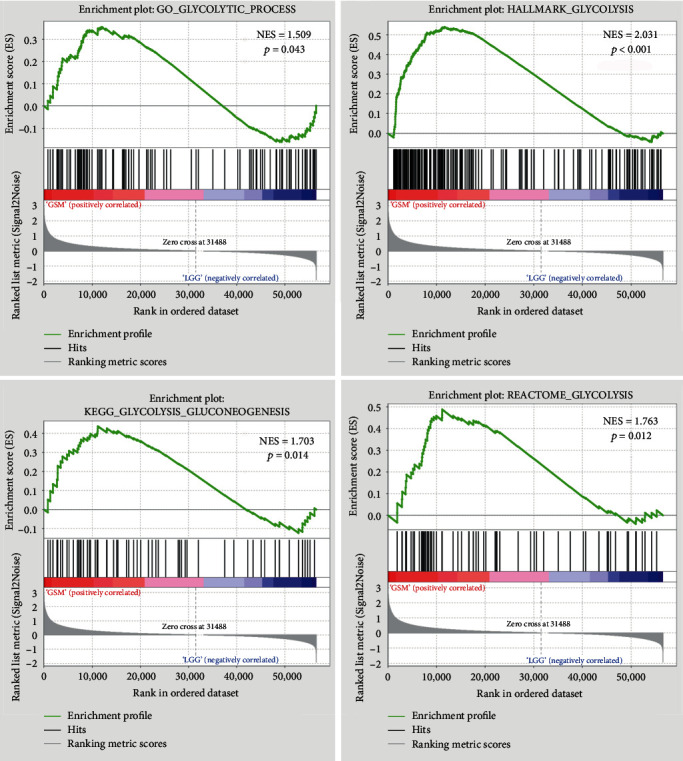
GSEA results of the enrichment plots of four gene sets (GO_GLYCOLYTIC_PROCESS, HALLMARK_GLYCOLYSIS, KEGG_GLYCOLYSIS_GLUCONEOGENESIS, and REACTOME_GLYCOLYSIS) that were significantly differentiated in LGG and GBM samples based on TCGA.

The mRNA expression and clinical data of all patients were obtained from TCGA. We found all glucose metabolism-related gene sets (*n* = 19) in the Molecular Signatures Database (MSigDB) version 7.1 to represent well-defined glucose metabolism states or processes. GSEA was performed to identify whether the gene sets showed significant differences between the LGG and GBM samples. Ultimately, we found that four gene sets, including GO_GLYCOLYTIC_PROCESS, HALLMARK_GLYCOLYSIS, KEGG_GLYCOLYSIS_GLUCONEOGENESIS, and REACTOME_GLYCOLYSIS, were significantly enriched with normalized *p* values < 0.05 (Figure 1). We then selected the four gene sets, which contained 327 specific genes for further analysis.

### 3.2. Glucose Metabolism-Related Gene-Based Prognostic Model

**Figure 2:**
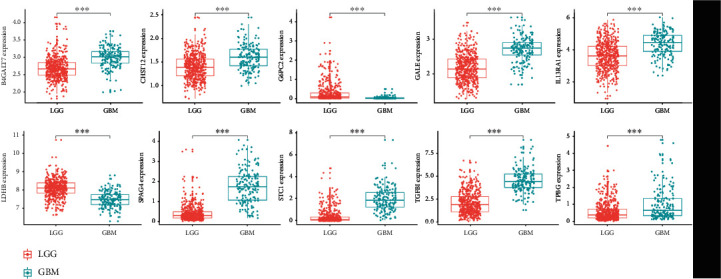
Different expressions of ten mRNAs in the LGG (*n* = 529) and GBM (*n* = 167) samples from The Cancer Genome Atlas (∗∗∗*p* < 0.001).

To identify novel genetic biomarkers associated with the outcomes of patients with GBM, we applied univariate Cox proportional hazards regression to 327 genes that were enriched in the four gene sets mentioned above. Twenty-seven genes were significantly correlated with OS (*p* < 0.05) and were included in a stepwise multivariate Cox regression analysis. Among the 27 genes, some were not significant in multivariate regression, and it was common in regression analysis because the potential correlation within variables may cause the *p* value to not be significant in multivariable Cox regression analysis. However, we still accept the predictive ability of these 27 genes. According to the Akaike information criterion [22], we achieved a compromise between variables and the accuracy of the regression model during the regression variable selection. Ultimately, ten independent genes (B4GALT7, CHST12, G6PC2, GALE, IL13RA1, LDHB, SPAG4, STC1, TGFBI, and TPBG) (Table 1) were selected via multivariable Cox regression analysis in R. Finally, a gene-based prognostic model was established to evaluate the survival risk of each patient as follows: risk score = expression of B4GALT7 × 2.0604 + expression of CHST12 × 1.4322 + expression of G6PC2 × (−2.8374) + expression of GALE × 1.4081 + expression of IL13RA1 × 1.0801 + expression of LDHB × (−3.2119) + expression of SPAG4 × 0.3957 + expression of STC1 × 0.4413 + expression of TGFBI × (−1.4198) + expression of TPBG × 0.5223. Then, the differential expression of the ten genes in LGG and GBM samples was also investigated. Eight genes (B4GALT7, CHST12, GALE, IL13RA1, SPAG4, STC1, TGFBI, and TPBG) were significantly upregulated in GBM samples, and two genes (G6PC2 and LDHB) were significantly upregulated in LGG samples (*p* < 0.0001, Figure 2).

### 3.3. Association between the Risk Score and Outcome of Patients

**Figure 3:**
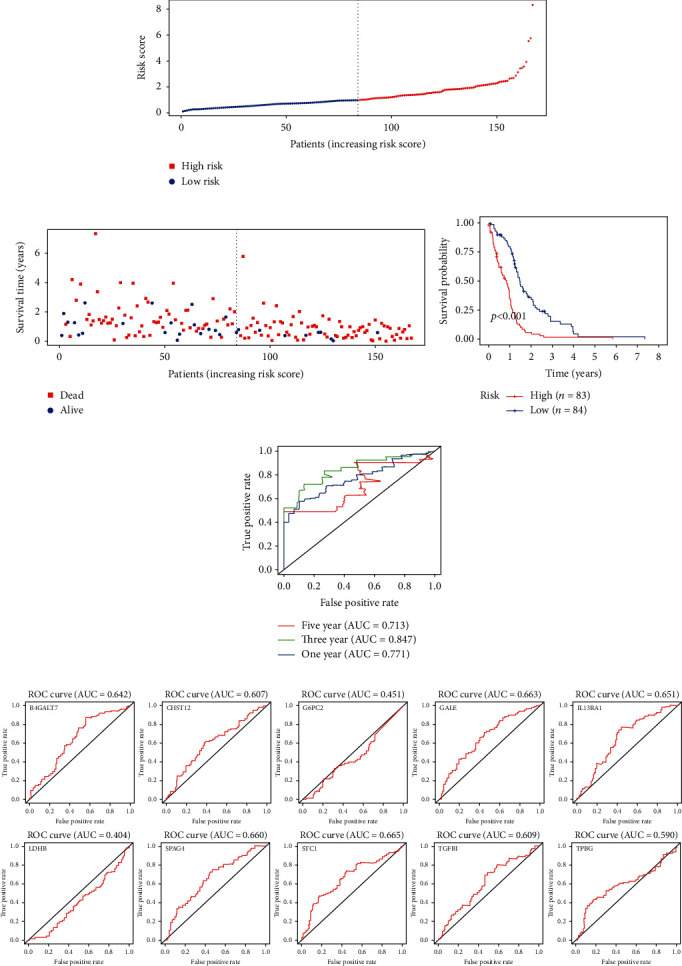
The ten-mRNA signature related to the risk score predicts the overall survival of patients with GBM: (a) risk score distribution; (b) survival status; (c) Kaplan-Meier survival curves showed the prognostic value of the risk signature between the low-risk group (*n* = 84) and the high-risk group (*n* = 83); (d) ROC curves were used to assess the efficiency of the risk signature for predicting 1-, 3-, and 5-year survival; (e) verifying that the prognostic value of the risk signature is better than that of each single biomarker with ROC curves.

The expressions of the ten genes were extracted from the transcriptome and substituted into the ten-mRNA signature, the risk scores for each patient with GBM were then calculated and ranked in order of increasing risk scores (Figure 3(a)) [23].  Figure 3(b) shows the risk score, OS (in years), and life status of 167 patients in the GBM dataset, ranked in order of increasing risk scores. Patients with high-risk scores had higher death rates than patients with low-risk scores. Then, the 167 patients in the entire GBM dataset were classified into the high-risk group (*n* = 83) and the low-risk group (*n* = 84) using the median risk score as the threshold. The KM analysis showed a significant difference in the outcomes of patients in the high-risk group and the low-risk group (log-rank test *p* < 0.001; Figure 3(c)). Patients in the high-risk group had a significantly worse survival than those in the low-risk group. To evaluate the efficiency of the ten-mRNA signature in predicting prognosis, receiver operating characteristic (ROC) curve analysis was carried out. The area under the curve (AUC) for the ten-mRNA signature was 0.771, 0.847, and 0.713 for one-year, three-year, and five-year survival, respectively (Figure 3(d)), demonstrating the reliable prognostic performance of the ten-mRNA signature for predicting survival in the entire dataset. To confirm that the gene signature performs better than the single gene biomarkers, we performed KM and ROC curve analyses, and the results supported our hypothesis. When the ten genes were each taken as a separate biomarker, their prognostic performance was not better than that of the ten-mRNA signature (Figure 3(e)).

### 3.4. The Risk Score Calculated through the Ten-mRNA Signature Is an Independent Prognostic Indicator

To investigate whether the risk score and clinical variables can predict patient survival, we performed univariate and multivariate Cox proportional hazards analyses, which included risk score, gender, age, KPS score, radiotherapy, chemotherapy, IDH status and MGMT promoter methylation status as covariables, to evaluate the potential of these indicators in the patient cohort. The results demonstrated that risk score (HR: 2.357; 95% confidence interval [CI]: 1.734–3.204; *p* < 0.001), age (HR: 2.576; 95% CI: 1.515–4.379; *p* < 0.001), radiotherapy (HR: 0.298; 95% CI: 0.160–0.555; *p* < 0.001), chemotherapy (HR: 0.488; 95% CI: 0.267–0.892; *p* = 0.020), IDH status (HR: 0.229; 95% CI: 0.056–0.943; *p* = 0.041), and MGMT promoter methylation status (HR: 0.539; 95% CI: 0.316–0.920; *p* = 0.023) were associated with patient survival in the univariate analysis. Additionally, risk score, age, and radiotherapy had dominant independent prognostic value both in the univariate analysis and in the multivariate analysis (*p* < 0.05), which proves that the prognostic value of the ten-gene signature is significant for survival prediction. These results demonstrated that the risk score was powerful in predicting the prognosis of patients with GBM (Table 2).

### 3.5. Validation of the Risk Signature

**Figure 4:**
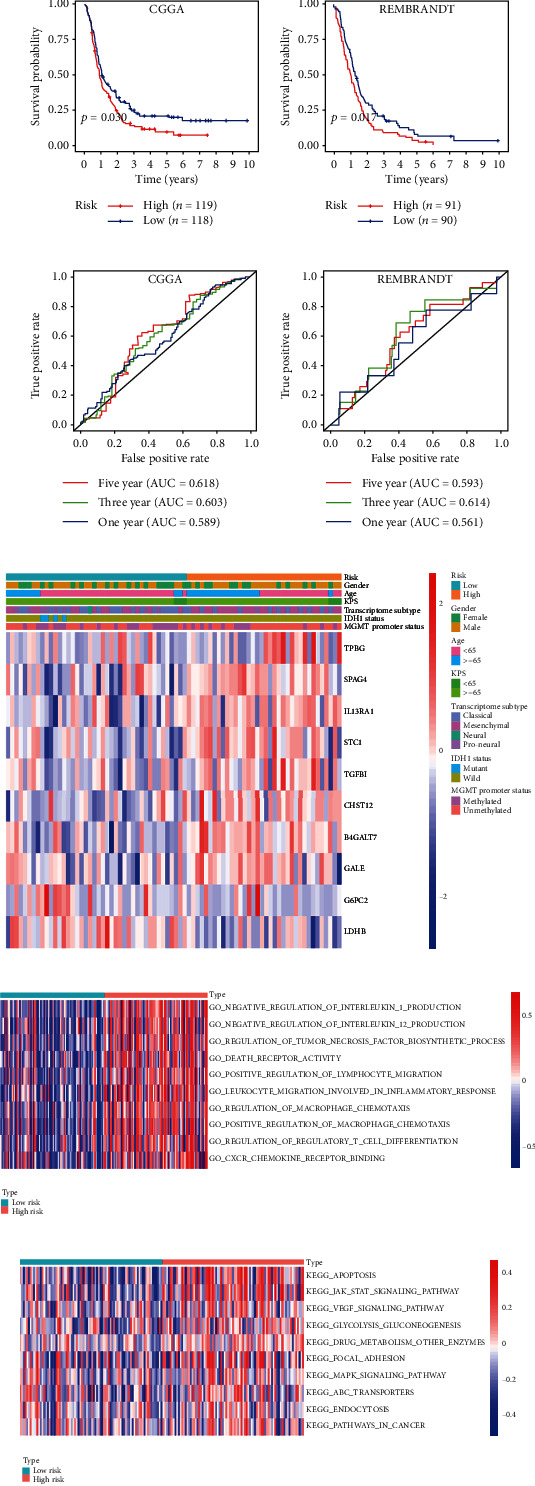
Evaluating the efficiencies of the risk signature in the CGGA and REMBRANDT datasets. (a, b) Kaplan-Meier survival curves showed the prognostic value of the risk signature in the CGGA dataset ((a) low-risk group, *n* = 118; high-risk group, *n* = 119; *p* < 0.05) and REMBRANDT dataset ((b) low-risk group, *n* = 91; high-risk group, *n* = 90; *p* < 0.01). (c, d) ROC curves evaluated the efficiency of the risk signature for predicting 1-, 3-, and 5-year survival in the (c) CGGA dataset and the (d) REMBRANDT dataset. Associations between the signature risk score and clinical features. (e) The heat map shows the associations between the risk signature and clinical characteristics (gender, age, KPS score, transcriptome subtype, IDH1 status, and MGMT promoter status) in the TCGA database. Functional roles of the risk signature. (f, g) GSVA showed the (f) biological processes and the (g) KEGG pathways associated with the risk signature.

We collected 237 GBM samples in the CGGA dataset and 181 GBM samples in the REMBRANDT dataset as two validation datasets to verify the excellent performance of the risk signature model. The KM survival curves indicated that patients with higher risk scores had a poorer prognosis than those with lower risk scores (Figure 4(a), CGGA, *p* < 0.05; and Figure 4(b), REMBRANDT, *p* < 0.05). The AUCs of the ROC curves for predicting the 1-, 3-, and 5-year survival of GBM patients in the CGGA dataset were 0.589, 0.603, and 0.618, respectively (Figure 4(c)), and those in the REMBRANDT dataset were 0.561, 0.614, and 0.593 (Figure 4(d)). These results showed that the risk signature performed well in predicting the survival of the GBM patients.

### 3.6. Associations between the Risk Signature and Clinical Variables

To explore the associations between the risk signature and clinical variables, we first present the distribution trends of gender, age, KPS score, transcriptome subtype, IDH1 status and MGMT promoter methylation status between the low-risk and high-risk groups in the TCGA database. As shown in Figure 4(e), the high-risk group tended to have more patients older than 65 years, whereas samples with IDH1 mutations were all included in the low-risk group, and samples with different transcriptome subtypes seemed to have distinct distributions in the two risk groups. Meanwhile, there was no apparent difference between the low-risk and high-risk groups in gender, KPS score, and MGMT promoter status. To be more intuitive, the chi-square test was used to verify the proportion differences of each factor (age, gender, molecular subtypes, MGMT promoter methylation status, and IDH1 status) between the low-risk and high-risk groups (Table 3). The results demonstrated that patients older than 65 years had more high-risk proportions, patients with wild-type IDH1 GBM had more high-risk proportions, and patients with GBM of the mesenchymal subtype had the highest high-risk proportions (Bonferroni's correction).

### 3.7. Functional Analysis associated with the Risk Signature

We used GSVA to investigate the biological processes and KEGG pathways related to the risk signature. As shown in Figure 4(f), several biological processes relevant to necrosis, leukocyte migration involved in the inflammatory response, positive regulation of macrophage chemotaxis, and regulatory T cell differentiation were enriched in the high-risk group. Regarding KEGG pathways, the high-risk group was positively correlated with apoptosis, focal adhesion, the MAPK and JAK-STAT signaling pathways, the VEGF signaling pathway, ABC transporters, and so on (Figure 4(g)). In short, these results revealed that the risk signature was correlated with almost every step of cancer progression.

### 3.8. Analysis Results of Differentially Expressed Genes in the Risk Signature

**Figure 5:**
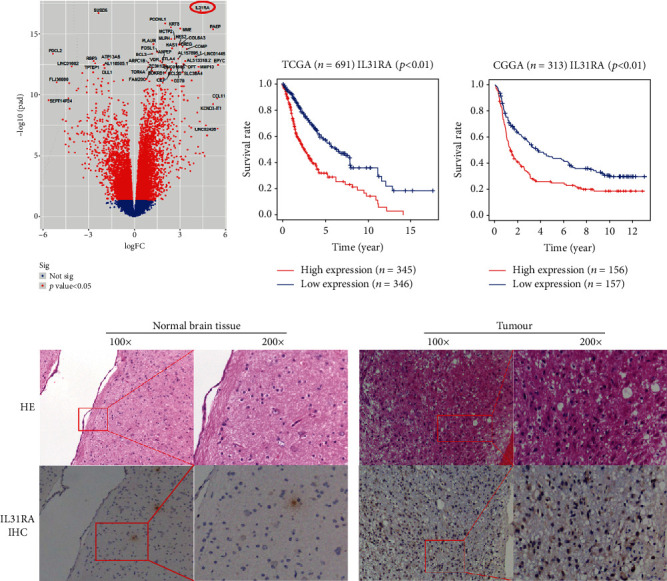
(a) Volcano map showing the differentially expressed genes between the high-risk group and the low-risk group. (b, c) Survival curve analysis of IL31RA expression based on the TCGA and CCGA databases. (d) Immunohistochemical expression of IL31RA in tumour and normal tissues.

The EdgeR method was used to analyse the differentially expressed genes between the high-risk group and the low-risk group, and then the volcano map was drawn. The results showed that IL31RA, PODNL1, KRT8, and other genes were positively correlated with the risk score of the model (*p* < 0.05), with IL31RA showing the strongest correlation, while JAZF1-ASI, FREM3, RHBG, and the rest of the genes were negatively correlated with the risk score of the model (*p* < 0.05) (Figure 5(a)). The transcriptome data and clinical data of 691 and 313 patients with gliomas were downloaded from the TCGA and CCGA databases, respectively. The survival time and survival status of the patients were extracted. The survival difference between patients with high and low expression of IL31RA was analysed by a KM curve. The results showed that IL31RA expression was negatively correlated with survival time (*p* < 0.05) (Figures 5(b) and 5(c)). Our immunohistochemical results showed that IL31RA was positively expressed in tumour tissues but not in normal brain tissues (Figure 5(d)).

## 4. Discussion

Currently, cancer research on energy metabolism has attracted much attention. In contrast to some other tumour types, aberrant glucose metabolism is an important component of GBM growth and chemoresistance [24]. Regulators of GBM glucose metabolism have been demonstrated to be useful tools for prognostication, diagnosis, and therapy [25]. However, due to the high heterogeneity of GBM, it is difficult for these biomarkers to independently and accurately predict the survival rate of patients. Therefore, in this study, we constructed a statistical model containing multiple glucose metabolism-related genes and combined the function of each gene to improve the prediction efficiency. This kind of model has been confirmed in many other solid tumours and is superior to a single biomarker in predicting tumour prognosis [26–28].

Ten glucose metabolism-related biomarker genes (B4GALT7, CHST12, G6PC2, GALE, IL13RA1, LDHB, SPAG4, STC1, TGFBI, and TPBG) were found to be statistically and biologically significant in the discrimination of LGGs from GBM in this study (Table 1). Among these biomarker genes, GALE encodes UDP-galactose-4-epimerase, which catalyses two distinct but analogous reactions: the epimerization of UDP-glucose to UDP-galactose and the epimerization of UDP-N-acetylglucosamine to UDP-N-acetylgalactosamine. GALE plays an important role in promoting the development of human glioma [29]. IL-13 receptor subunits *α*1 and *α*2 of the IL-13R complex are overexpressed in GBM. Jing Han and his colleagues showed that high IL-13R*α*1 with or without IL-13R*α*2 expression was associated with poor prognosis in patients with high-grade gliomas. Nevertheless, there was no correlation between IL-13R*α*1 and IL-13R*α*2 mRNA expression. Their findings have important implications in understanding the role of IL-13R in the pathogenesis of GBM and potentially other cancers [30]. Further, IL-13R*α*2 and IL4R may also play an important role in the polygenic prognostic risk signature due to their potential functional association with IL-13R*α*1 in the future. LDHB is a dehydrogenase and a critical switch that regulates glycolysis and OXPHOS. It has been proven that the expression of LDHB alone was not able to predict a difference in OS, but the concomitant expression of LDHB and CCNB1 was able to identify medulloblastoma patients with a significantly worse prognosis [31]. SPAG4 is a member of the cancer testis (CT) gene family and to date, little is known about its physiological function or its involvement in tumour biology, but there is a research that it is a potential marker in glioma [32, 33]. STC1 encodes a secreted, homodimeric glycoprotein that is expressed in a wide variety of tissues and may have autocrine or paracrine functions, STC1 is a novel noncanonical NOTCH ligand and acts as a crucial regulator of stemness in GBM [34]. Transforming growth factor-beta-induced (TGFBI) is an exocrine protein that has been proven to promote the development of glioma, nasopharyngeal carcinoma, bladder cancer and other tumours [35, 36]. In a recent study, Guo Sk and colleagues showed that TGFBI was upregulated in glioma cells and played a promoting role in the growth and motility of U87 and U251 cells. Their results suggested that TGFBI has the potential to be a diagnostic marker and to serve as a target for the treatment of gliomas [37]. In addition, there is no research on B4GALT7, CHST12, G6PC2 and TPBG in glioma. Although these genes can independently predict tumour prognosis to some extent, our results demonstrated that the ten-mRNA signature has better prognostic significance than the corresponding single biomarkers. Moreover, by using KM and ROC curve analyses of GBM, we verified our statistical results in the CGGA and REMBRANDT datasets. We confirmed that the risk signature performed well in predicting the survival of patients with GBM (Figures 4(a)–4(d)). Therefore, this glucose metabolism-related gene signature can predict tumour prognosis more accurately and guide treatment more comprehensively.

We also constructed a heat map to present the associations between the risk signature and clinical characteristics in the TCGA database. Our results indicated that elderly age, mesenchymal subtype, and wild-type IDH1 were significantly correlated with higher risk scores (Table 3, Figure 4(e)). Consistent with mainstream views, elderly patients, the mesenchymal subtype, and wild-type IDH1 usually predict an unfavourable prognosis [38, 39]. Moreover, by using GSVA to explore the biological processes and KEGG pathways associated with the risk signature, we noticed that the risk signature was correlated with almost every step of oncogenesis and tumour progression, including adverse biological processes and signal transduction pathways (Figures 4(f) and 4(g)). Currently, many studies have elucidated the aggressive behaviours associated with GBM glucose metabolism and attempted to find ways to target GBM glucose metabolism, such as through Myc, PGK1, SIRT3, and HK1 [40–43]. Therefore, our results once again confirm the reliability of the risk score in predicting the prognosis of GBM and provide new potential targets for targeting glucose metabolism.

A few reports in the literature have recently implicated the IL-31/IL31RA axis in cancer [44, 45]. However, the role and mechanism of IL31RA in glioma progression are still unclear. Our results show a significant difference in IL31RA expression between the high- and low-risk groups, and the TCGA/CCGA database shows that the higher expression of IL31RA is significantly associated with the poor prognosis of GBM patients. Moreover, we found that IL31RA expression was positive in tumour tissue but negative in normal brain tissue. Therefore, IL31RA is expected to be a potential therapeutic target in glioma.

## 5. Conclusions

In conclusion, we identified and validated a risk signature with ten glucose metabolism-related genes associated with the survival of patients with GBM, where higher risk scores indicate unfavourable outcomes. Moreover, based on the signature, we found that a different gene, IL31RA, may be a potential therapeutic target in GBM. Our findings may provide novel insights for GBM research and guidance for individual therapy.

## Figures and Tables

**Table 1:** The detailed information of ten prognostic mRNAs which were selected via multivariable Cox regression analysis.

mRNA	Ensemble ID	Location	*β* (Cox)	HR (95% CI)	*p* value
B4GALT7	ENSG00000027847	Chr5: 177,600,102-177,610,330	2.0604	7.8488 (0.8616-71.5017)	0.0676
CHST12	ENSG00000136213	Chr7: 2,403,489-2,448,484	1.4322	4.1879 (1.1431-15.3426)	0.0306∗
G6PC2	ENSG00000152254	Chr2: 168,901,223-168,910,000	−2.8374	0.0586 (0.0069-0.4932)	0.0091∗∗
GALE	ENSG00000117308	Chr1: 23,795,599-23,800,754	1.4081	4.0882 (0.9346-17.8829)	0.0615
IL13RA1	ENSG00000131724	ChrX: 118,726,954-118,794,533	1.0801	2.9449 (1.1305-7.6718)	0.0270∗
LDHB	ENSG00000111716	Chr12: 21,635,342-21,657,971	−3.2119	0.0403 (0.0020-0.7950)	0.0348∗
SPAG4	ENSG00000061656	Chr20: 35,615,829-35,621,094	0.3957	1.4853 (0.9105-2.4232)	0.1131
STC1	ENSG00000159167	Chr8: 23,841,929-23,854,806	0.4413	1.5547 (0.9410-2.5686)	0.0849
TGFBI	ENSG00000120708	Chr5: 136,028,988-136,063,818	−1.4198	0.2418 (0.1027-0.5689)	0.0011∗∗
TPBG	ENSG00000146242	Chr6: 82,362,983-82,367,420	0.5223	1.6858 (1.1448-2.4826)	0.0082∗∗

**Table 2:** Univariable and multivariable analyses for each clinical feature.

Clinical feature	Univariate analysis			Multivariate analysis		
	HR	95% CI of HR	*p* value	HR	95% CI of HR	*p* value
Risk score (low-risk/high-risk)	2.357	1.734-3.204	<0.001∗∗∗	1.822	1.252-2.651	0.002∗∗
Gender (female/male)	1.332	0.788-2.250	0.284	1.167	0.592-2.299	0.655
Age (<65/≥65)	2.576	1.515-4.379	<0.001∗∗∗	2.270	1.274-4.044	0.005∗∗
KPS (<60/≥60)	0.983	0.964-1.002	0.082	0.984	0.960-1.009	0.215
Radiotherapy (untreated/treated)	0.298	0.160-0.555	<0.001∗∗∗	0.348	0.131-0.926	0.035∗
Chemotherapy (untreated/treated)	0.488	0.267-0.892	0.020∗∗	1.468	0.559-3.855	0.436
IDH status (wild/mutant)	0.229	0.056-0.943	0.041∗	0.583	0.122-2.793	0.499
MGMT promoter status (unmethylated/methylated)	0.539	0.316-0.920	0.023∗	0.879	0.457-1.689	0.698

**Table 3:** Associations between the signature risk score and clinical features.

Clinical feature	Risk score		*X* ^2^	*p*
	High-risk *n* (%)	Low-risk *n* (%)		
*Gender*			0.011	0.917
Female	29(49.15%)	30(50.85%)		
Male	54(50.00%)	54(50.00%)		
*Age*			3.974	0.046∗
<65	47(43.93%)	60(56.07%)		
≥65	36(60.00%)	24(40.00%)		
*KPS*	Fisher's exact test			0.572
<60	3(42.86%)	4(57.14%)		
≥60	55(47.01%)	62(52.99%)		
*Transcriptome subtype*		Fisher's exact test		<0.0001∗∗∗∗
Classical	20(38.46%)	32(61.54%)		
Mesenchymal	54(73.97%)	19(26.03%)		
Neural	4(57.14%)	3(42.86%)		
Proneural	1(5.26%)	18(94.74%)		
*IDH1 status*			7.669	0.006∗∗
Wild	78(52.35%)	71(47.65%)		
Mutant	1(9.09%)	10(90.91%)		
*MGMT promoter status*			1.271	0.260
Unmethylated	37(50.00%)	37(50.00%)		
Methylated	22(40.00%)	33(60.00%)		

## Data Availability

All original data for model building was downloaded from The Cancer Genome Atlas (TCGA) database (https://cancergenome.nih.gov/) and all original data for model validation was downloaded from the Repository for Molecular Brain Neoplasia Data (REMBRANDT, microarray) and Chinese Glioma Genome Atlas (CGGA, microarray) datasets which were downloaded from GlioVis (http://gliovis.bioinfo.cnio.es/). All authors appreciate the three abovementioned databases for this study.
